# Supplementary material for: Seven centuries of reconstructed Brahmaputra River discharge demonstrate underestimated high discharge and flood hazard frequency
Source: Nat Commun. 2020 Nov 26;11:6017. doi: 10.1038/s41467-020-19795-6 (PMC7692521; doi:10.1038/s41467-020-19795-6)
Supplement: Supplementary file 1 — Supplementary Information [file 41467_2020_19795_MOESM1_ESM.pdf]

Supplementary Information for

Seven centuries of reconstructed Brahmaputra River discharge demonstrate underestimated high discharge and flood hazard frequency

Mukund P. Rao[1,2,*], Edward R. Cook [1], Benjamin I. Cook[3,4], Rosanne D. D'Arrigo[1], Jonathan G. Palmer[5], Upmanu Lall[6], Connie A. Woodhouse[7], Brendan M. Buckley[1], Maria Uriarte[8], Daniel A. Bishop[1,2], Jun Jian[9], and Peter J. Webster[10]

[1]Tree Ring Laboratory, Lamont-Doherty Earth Observatory of Columbia University, Palisades, NY 10964, USA; [2]Department of Earth and Environmental Science, Columbia University, New York, NY 10027, USA; [3]NASA Goddard Institute for Space Studies, New York, NY 10025, USA; [4]Ocean & Climate Physics, Lamont-Doherty Earth Observatory of Columbia University, Palisades, NY 10964, USA; [5]ARC Centre of Excellence in Australian Biodiversity and Heritage, School of Biological, Earth and Environmental Sciences, University of New South Wales, Sydney, New South Wales 2052, Australia; [6]Department of Earth and Environmental Engineering, Columbia University, New York, NY 10027, USA; [7]School of Geography and Development, University of Arizona, Tucson, AZ 85721, USA; [8]Ecology, Evolution, and Environmental Biology, Columbia University, New York, NY 10027, USA; [9]Dalian Maritime University, Dalian 116024, China; and [10]Earth and Atmospheric Sciences, Georgia Institute of Technology, Atlanta 30318, USA. *E-mail: mukund24rao@gmail.com & mukund@ldeo.columbia.edu

Supplementary figures

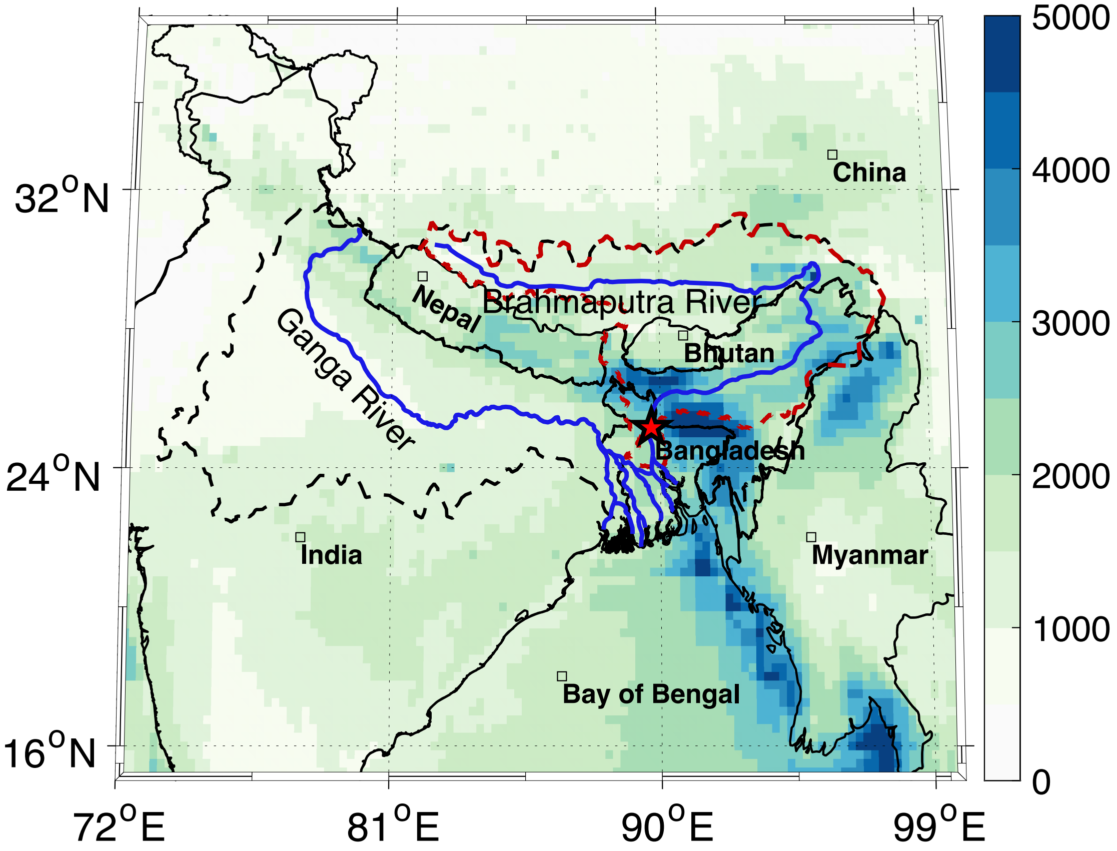

Supplementary Figure 1 Mean annual precipitation averaged between 2001 and 2015 as estimated by the TRMM dataset (mm/year) showing the high annual precipitation amounts in the Brahmaputra River watershed. The larger Ganga-Brahmaputra-Meghna watershed is demarcated as black dashed lines while the boundaries of the Brahmaputra watershed are demarcated using red dashed lines. The red star is the location of the Bahadurabad streamflow gauge in the Bangladesh. TRMM – Tropical Rainfall Monitoring Mission[1].

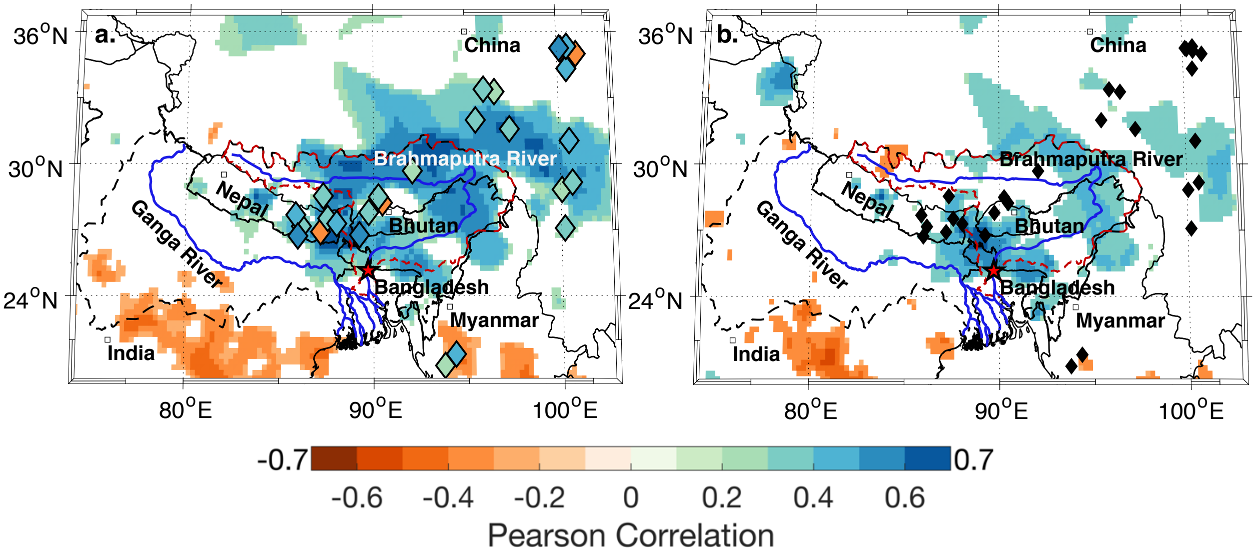

Supplementary Figure 2 Same as Main Text Figure 1 except that the shading on the map represents the spatial field correlation between **(a)** July-August-September (JAS) discharge at the Bahadurabad gauge and mean JAS precipitation from the GPCC v2018 dataset[2] between 1956-2011 C.E., and **(b)** the first principal component (PC1) of the 28 tree ring predictors (variance explained: 24.86%) and mean JAS GPCC precipitation (1956-1998 C.E.). These correlations are slightly weaker than those found in Figure 1 (main text) against CRU precipitation but are consistent with its both Brahmaputra JAS flow at Bahadurabad and our tree ring predictor network being sensitive to upper basin precipitation. The shading in the diamonds in **a.** represents the correlation of each tree-ring predictor series with mean JAS discharge at Bahadurabad between 1956-2011 C.E. These remain same as in Main Text Figure 1. Only correlations significant at $p<0.05$ using a 2-sided t-test are shown. Note that the locations of tree ring predictors are jittered for display. GPCC - Global Precipitation Climatology Centre.

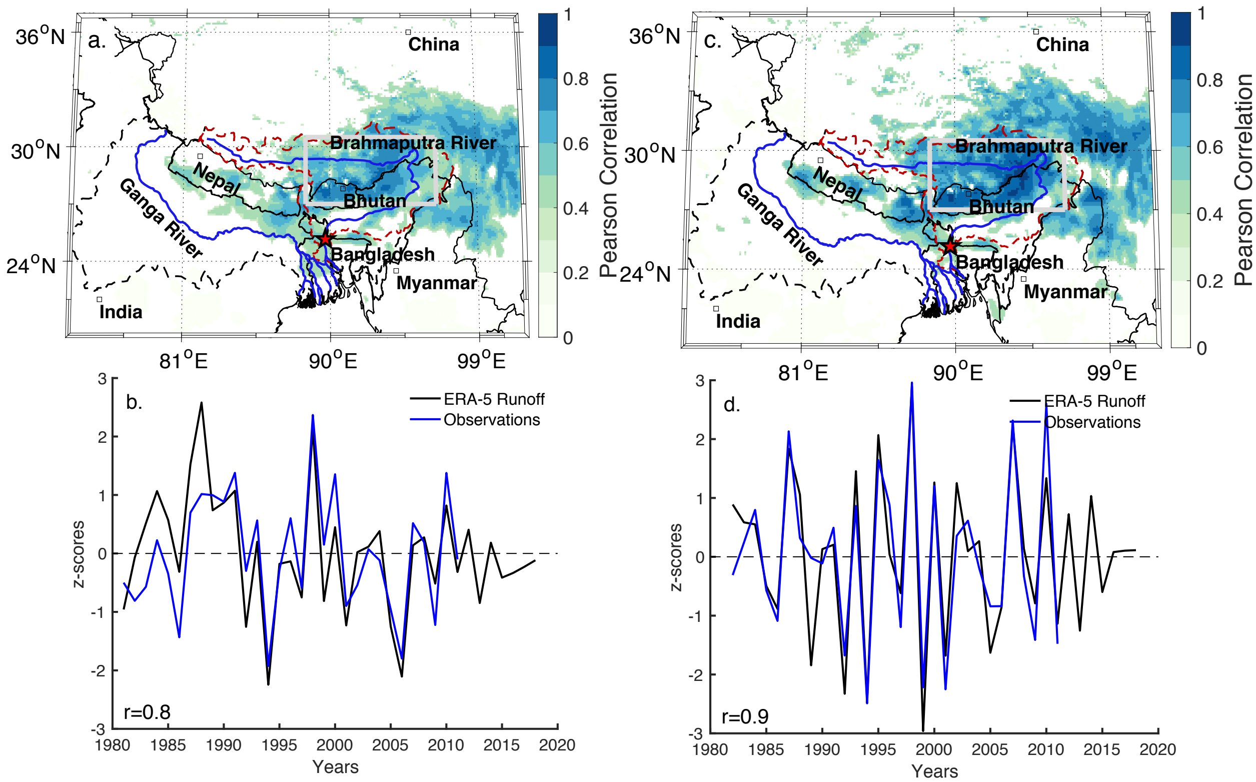

Supplementary Figure 3 Pearson correlation between mean July-August-September (JAS) discharge at the Bahadurabad gauge, Bangladesh, and ERA-5 reanalysis modelled JAS runoff between 1981-2011 C.E. (31 years). Correlations are calculated using discharge and modelled runoff data (**a & b**), and using first-differenced discharge and first-differenced runoff data (**c & d**). For the timeseries comparisons in **c** and **d** ERA-5 modelled runoff was averaged in the grey shaded box shown in **a** and **b** spanning the upper basin (88.5-96.5°E and 27-30.5°N). ERA-5 runoff is modelled as the sum of surface and sub-ground runoff and is driven primarily by precipitation, melting snow, and soil storage in the model formulation. The high correlations between discharge at Bahadurabad and independent estimates of runoff from a hydrologic model driven using climate variables validates the robustness of the discharge data and that lower basin discharge at Bahadurabad is driven by upper basin runoff (that is in turn controlled by upper basin precipitation - see Main Text Fig. 1 and Supplementary Fig. S2 and S4). Only correlations significant at $p<0.05$ using a 2-sided t-test are shown.

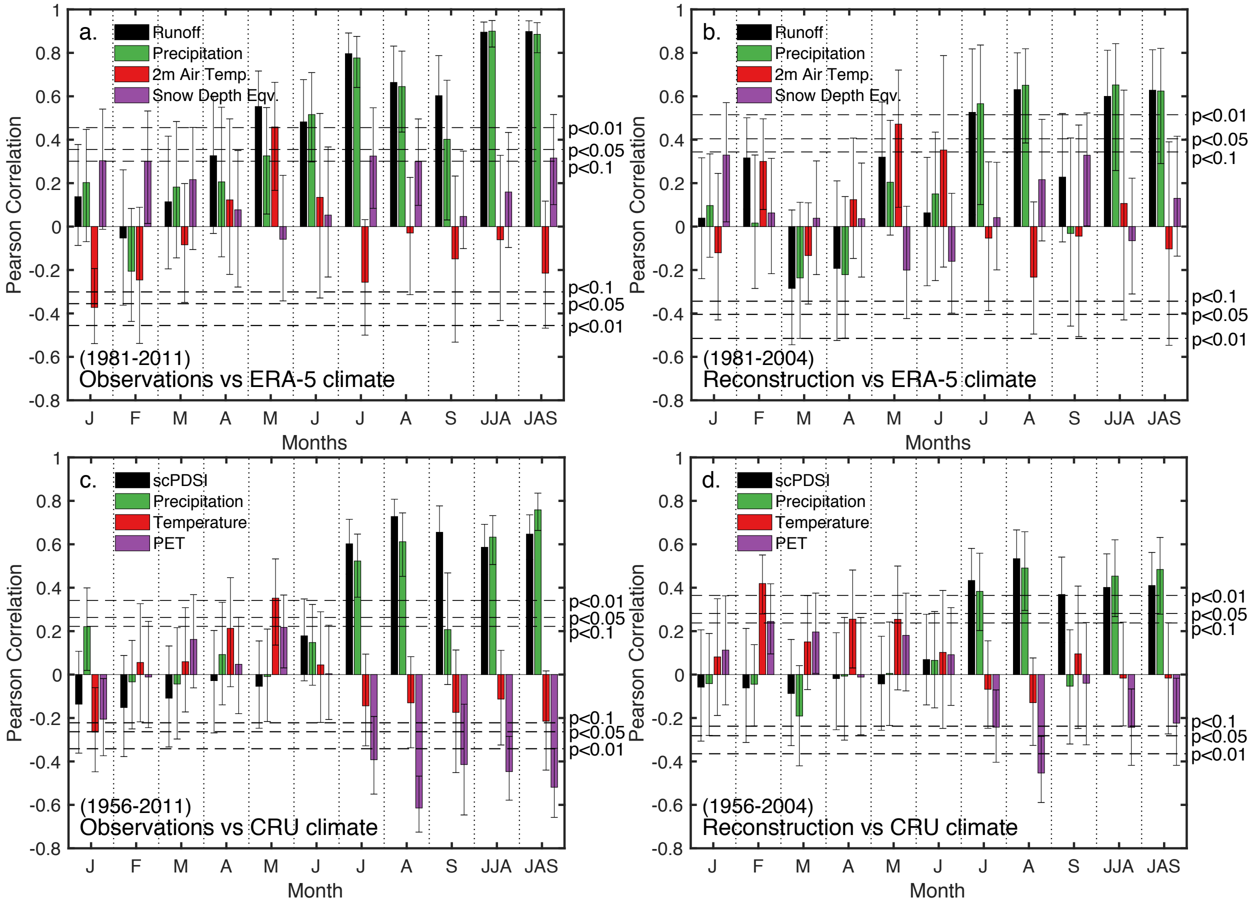

Supplementary Figure 4 Correlation response function plot for mean July-September (JAS) instrumental and reconstructed discharge of the Brahmaputra River at Bahadurabad, Bangladesh against upper basin (88.5-96.5°E and 27-30.5°N) monthly climate variables from the ERA-5 (parts **a-b**) CRU (parts **c-d**) datasets. This is the same region for which ERA-5 simulated runoff was averaged in Supplementary Figure S3 (grey box), to compare simulated upper basin runoff against observations of discharge at Bahadurabad, Bangladesh. The climate variables used from the ERA-5 dataset (parts **a-b**) include monthly simulated runoff, precipitation, 2m air temperature, and snow depth equivalent averaged over the upper basin. The climate variables from the CRU dataset include scPDSI, precipitation, temperature, and potential evapotranspiration (PET) also averaged over the upper basin. The left panels (**a and c**) are for instrumental observations between 1981-2011 and 1956-2011 respectively. The right panels (**b and d**) are for reconstructed discharge between 1981-2004 and 1956-2004 respectively. The six horizontal dashed lines in each plot indicate three different thresholds for each monthly correlation to be statistically significant using a 2-tailed t-test. The median correlation and the error bars around each correlation bar are computed from 1,000 bootstrapped draws with replacement from the observed/reconstructed discharge series and the climate series. The last two columns of each subplot are the correlation between mean JAS observed/reconstructed discharge and mean upper basin climate averaged between July-August (JJA) and JAS.

Both instrumental observations and the reconstruction of JAS discharge correlate significantly with upper basin precipitation in the months of June-August (JJA) and July-September (JAS) for the ERA-5 and CRU climate datasets ($p<0.01$). However, these relationships are weaker for reconstructed discharge than for instrumental discharge (right vs left panels). scPDSI - self calibrating Palmer Drought Severity Index[3].

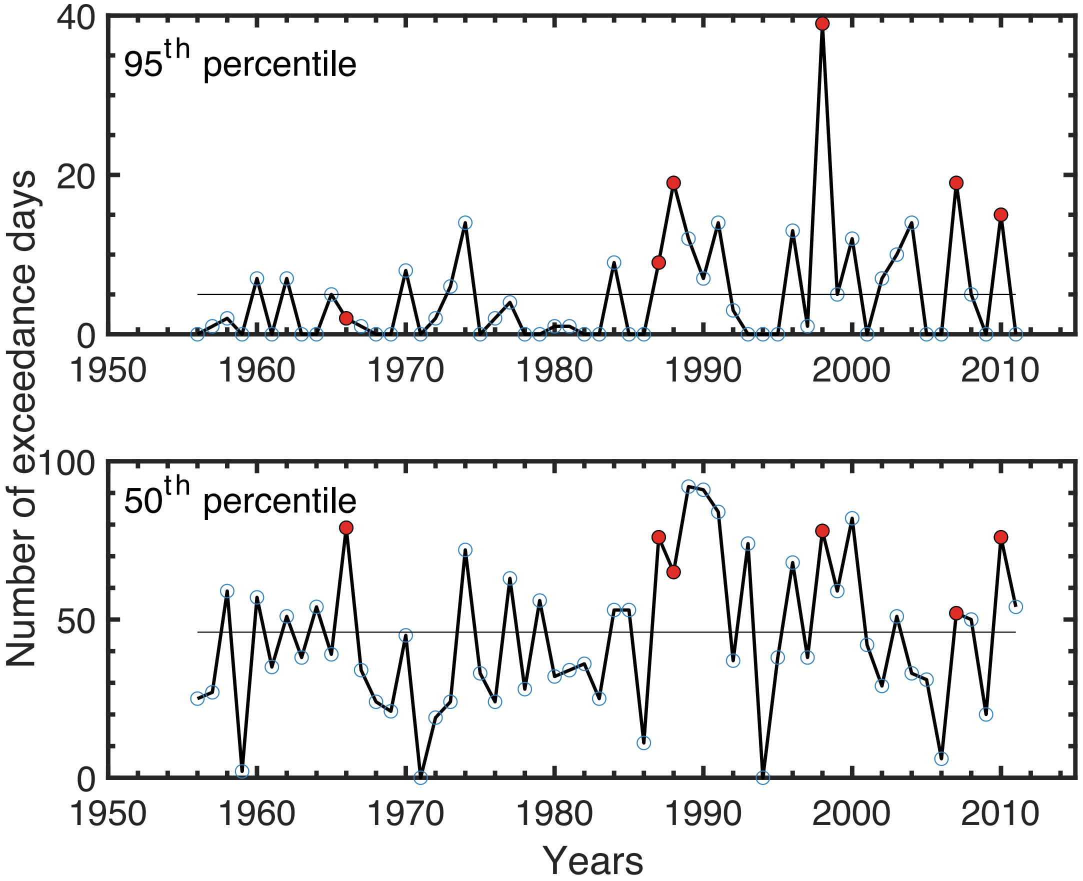

Supplementary Figure 5 Total number of days in July-August-September (JAS) in which daily flows at Bahadurabad exceeded the 95th percentile (top panel) and 50th percentile (lower panel) flow for the same day (1956-2011 C.E.). The 95th and 50th percentile daily flows were calculated between 1956 and 2011 C.E. The horizontal lines at 5 days and 46 days on the upper and lower panels represent 5% and 50% of the total of 92 days between July and September, and are the average exceedance days expected by chance in any given year. Red filled in circles indicate known instrumental period flood years in 1966, 1987, 1988, 1998, 2007, and 2010 C.E. Note that in 1966 C.E. daily flow never exceeded the 95th percentile of daily flow.

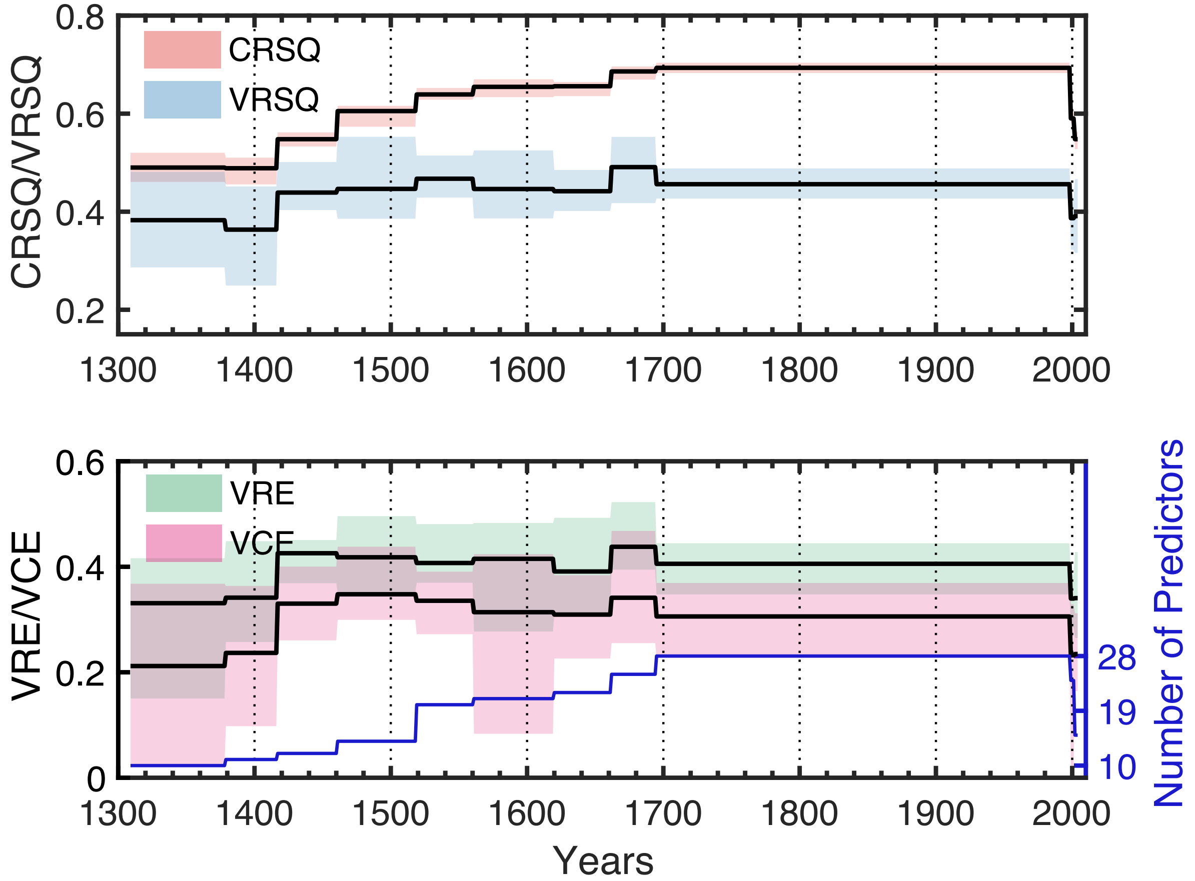

Supplementary Figure 6 Calibration and validation statistics of the mean JAS discharge reconstruction of the Brahmaputra River at Bahadurabad, Bangladesh along with the number of tree ring series used as predictors in each nest. The shaded uncertainties represent the range of variation in the statistic depending on the choice of correlation weight used in the Principal Components matrix weighting procedure (see Methods section). CRSQ - calibration period coefficient of multiple determination; VRSQ - validation period square of the Pearson correlation; VRE - validation period reduction of error; and VCE validation period coefficient of efficiency. VRE and VCE values consistently greater than 0 suggest reconstruction skill. The median value of each statistic is: i. CRSQ: 65.58%, ii. VRSQ:45.61 %, iii. VRE: 0.41, and iv. VCE: 0.31.

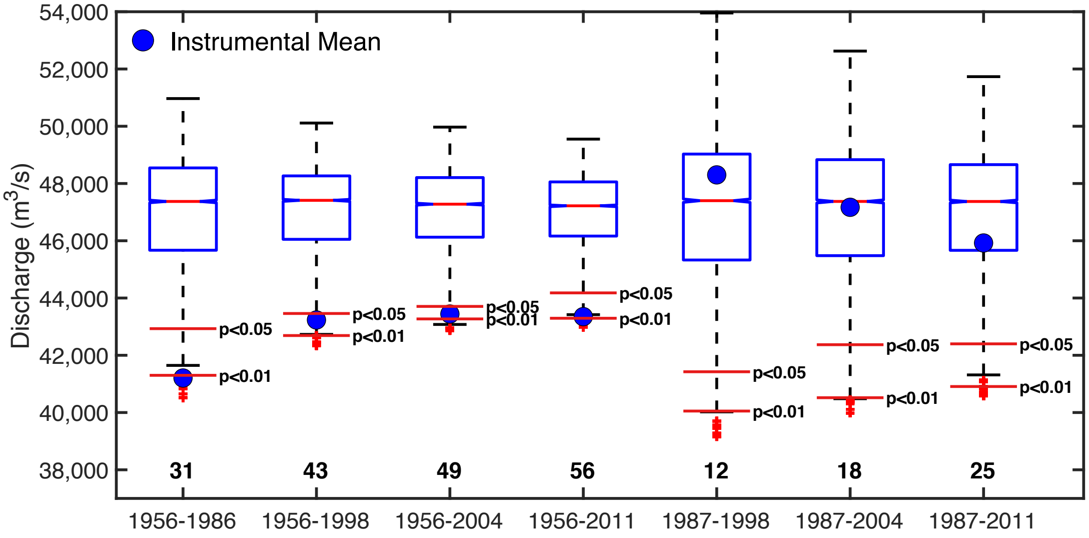

Supplementary Figure 7 Comparison between mean instrumental July-August-September (JAS) Brahmaputra River discharge at 7 different time intervals (1956-1986; 1956-1998; 1956-2004; 1956-2011; 1987-1998; 1987-2004; and 1987-2011) as filled blue dots against distributions of the mean reconstructed discharge in 10,000 random draws of blocks of same length from the reconstruction. The block length used in each draw is mentioned below each box plot. The two red horizontal lines indicate the threshold for mean discharge to be significantly drier than reconstructed discharge at $p<0.05$ and $p<0.01$. The plot suggests that the first 31 years of instrumental discharge between 1956-1986 were exceptionally dry ($p<0.05$) while discharge since 1987 C.E. aligns more closely with mean reconstructed discharge rates in the context of the past 7 centuries.

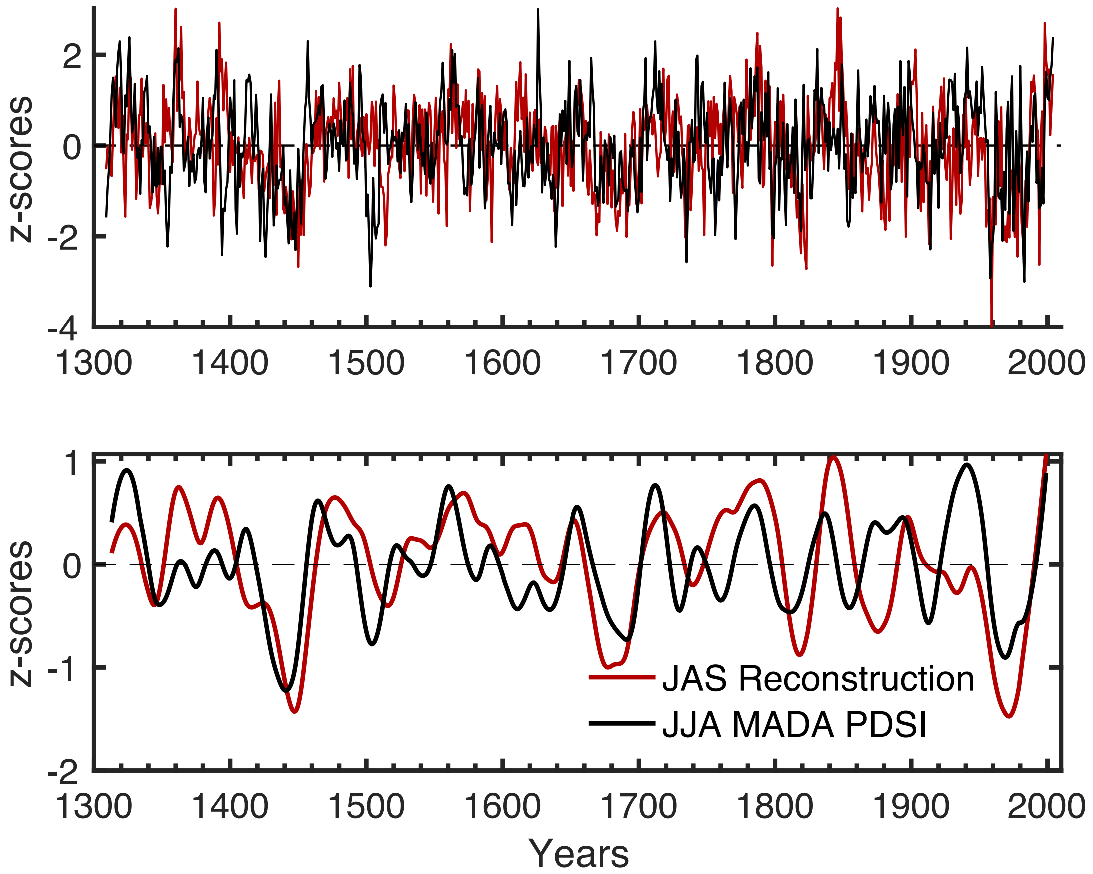

Supplementary Figure 8 A comparison between standardized versions of our JAS discharge reconstruction at Bahadurabad (in red) and a spatial average of the June-July-August (JJA) mean Palmer Drought Severity Index (PDSI) over the Brahmaputra watershed reconstructed by the Monsoon Asia Drought Atlas (MADA[4]) between 1309-2004 C.E. (Pearson $r=0.27$, $n=696$, $p<0.001$). While both datasets share many of the underlying predictors, they have different reconstruction target fields (Brahmaputra discharge vs gridded PDSI) and were produced using different reconstruction methods (Bayesian Regression vs spatial point-by-point regression). The lower panel compares 50-year low-pass filtered versions of both reconstruction highlighting that multi-decadal dry and wet periods over the basin suggested by our JAS reconstruction are also suggested by larger scale reconstructions of spatial drought variability. The 2 low-pass filtered series correlate at 0.52. While the low-pass filtered versions of the reconstructions show good visual correspondence, we note that this correlation is not 'statistically significant' at $p<0.05$ using a 2-tailed t-test considering the small effective sample size of 13.9 years (calculated as 696/50). The correlation needed for a $p<0.05$ for a sample size of 13.9 is 0.5342. The wet and dry periods we observe here also largely consistent with those found by refs. [5-8].

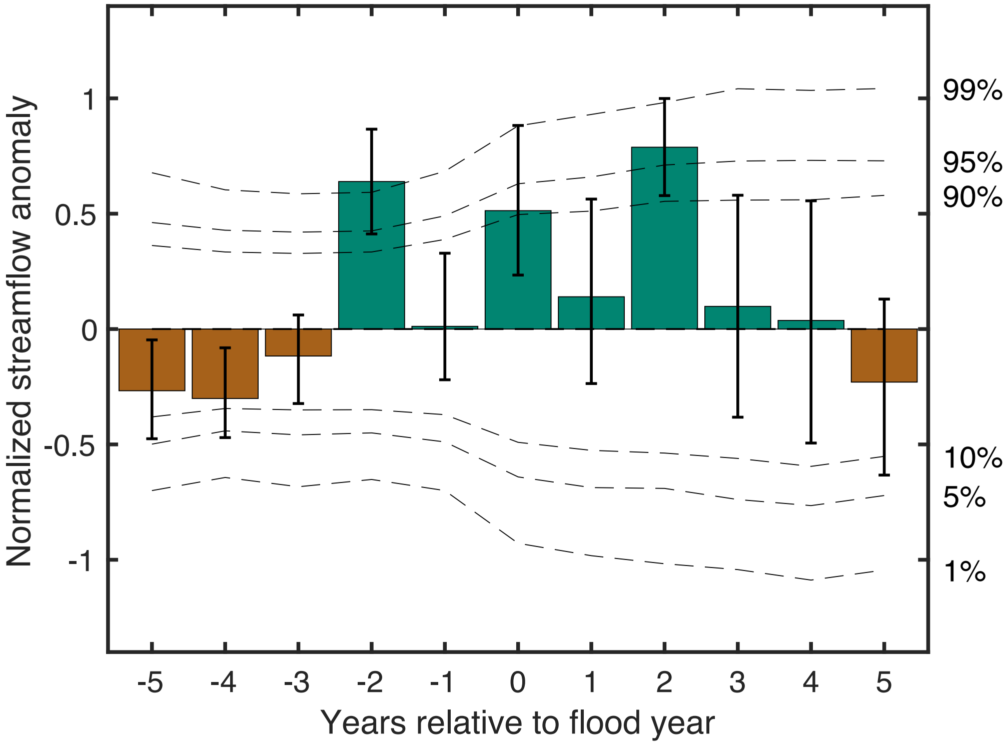

Supplementary Figure 9 Superposed Epoch Analysis (SEA) for discharge in 12 historical flood years that occurred prior to the start of instrumental observations in 1956 C.E. The vertical lines on the response bars are the 5th, 50th, and 95th percentiles of mean flow across 495 unique draws of 8 flood years at random out of 12. The horizontal dotted lines indicate the threshold required for epochal anomalies to be statistically significant using random bootstrapping at three different statistical thresholds. These thresholds were calculated by compositing 10,000 draws of 8 years at random (or 'pseudo-flood years') from the reconstruction between 1780 and 2004. The relationship between high discharge during flood years in much weaker than that for just the instrumental period flood years (Main Text, Fig. 2b) and for all 16 flood years (Main Text, Fig. 4a). The median response of the 495 unique draws of 8 flood years out of 12 is not significant at $p<0.05$ when compared to 10,000 draws of 8 'pseudo-flood' years at random.

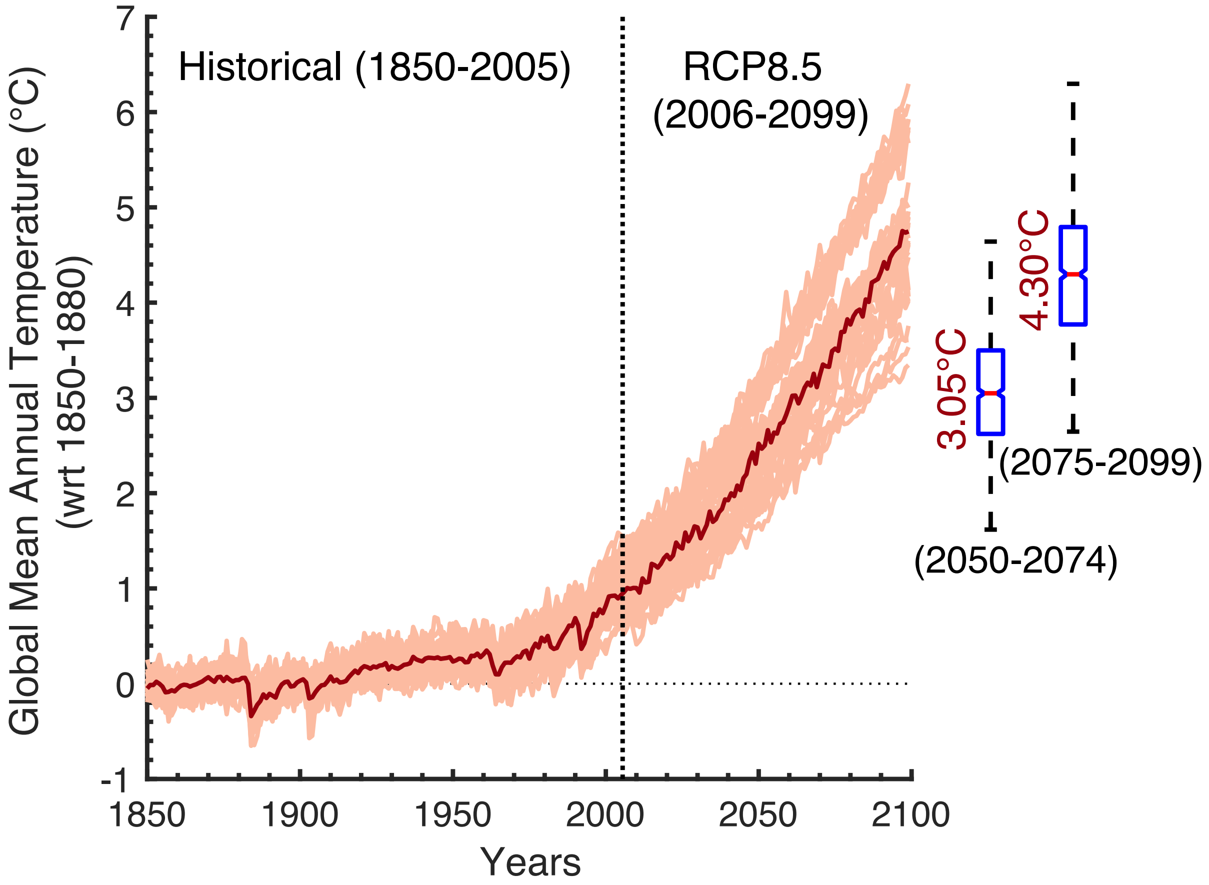

Supplementary Figure 10 Expected change in (area-weighted) global mean annual surface temperature between 2050-2074 C.E. and 2057-2099 C.E. relative to pre-industrial 1850-1880 C.E. mean conditions using CMIP-5 RCP8.5 projections. The multi-model median warming for these two periods is projected to be 3.05°C and 4.30°C respectively. We used the same suite of 20 models and 42 ensemble members an in our modelled runoff calculations for this analysis. The full list of models and the respective ensemble members can be found in Supplementary Table, S1.

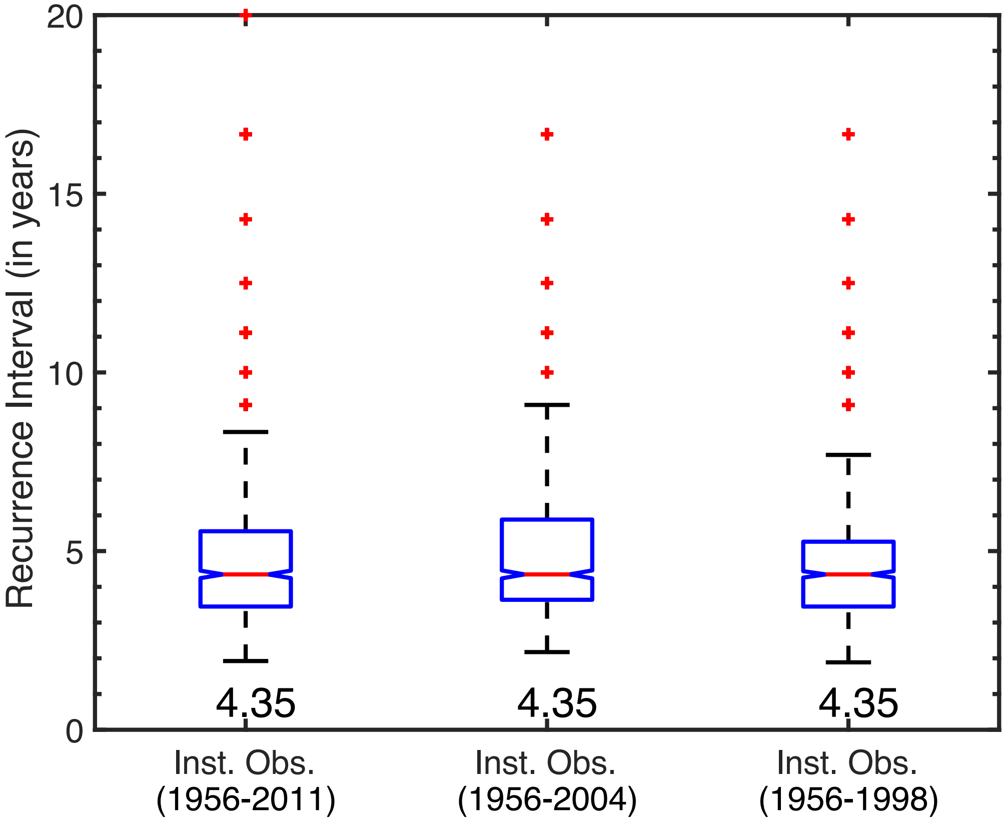

Supplementary Figure 11 Recurrence interval (in years) of discharge greater than the 2007 flood year in three different time periods of the observed instrumental data, i. 1956-2011 C.E., ii. 1956-2004 C.E., and iii. 1956-1998 C.E. The first period includes all instrumental observations, the second is the period of overlap between the instrumental observations and the reconstruction, and the third is the calibration-validation period for the reconstruction. The median recurrence interval is 4.35 for all three time periods, though there are slight differences in the range of variability across the 1,000 draws of 30-years with replacement. We note that the lack of difference in the median return interval could be in part due the short instrumental series.

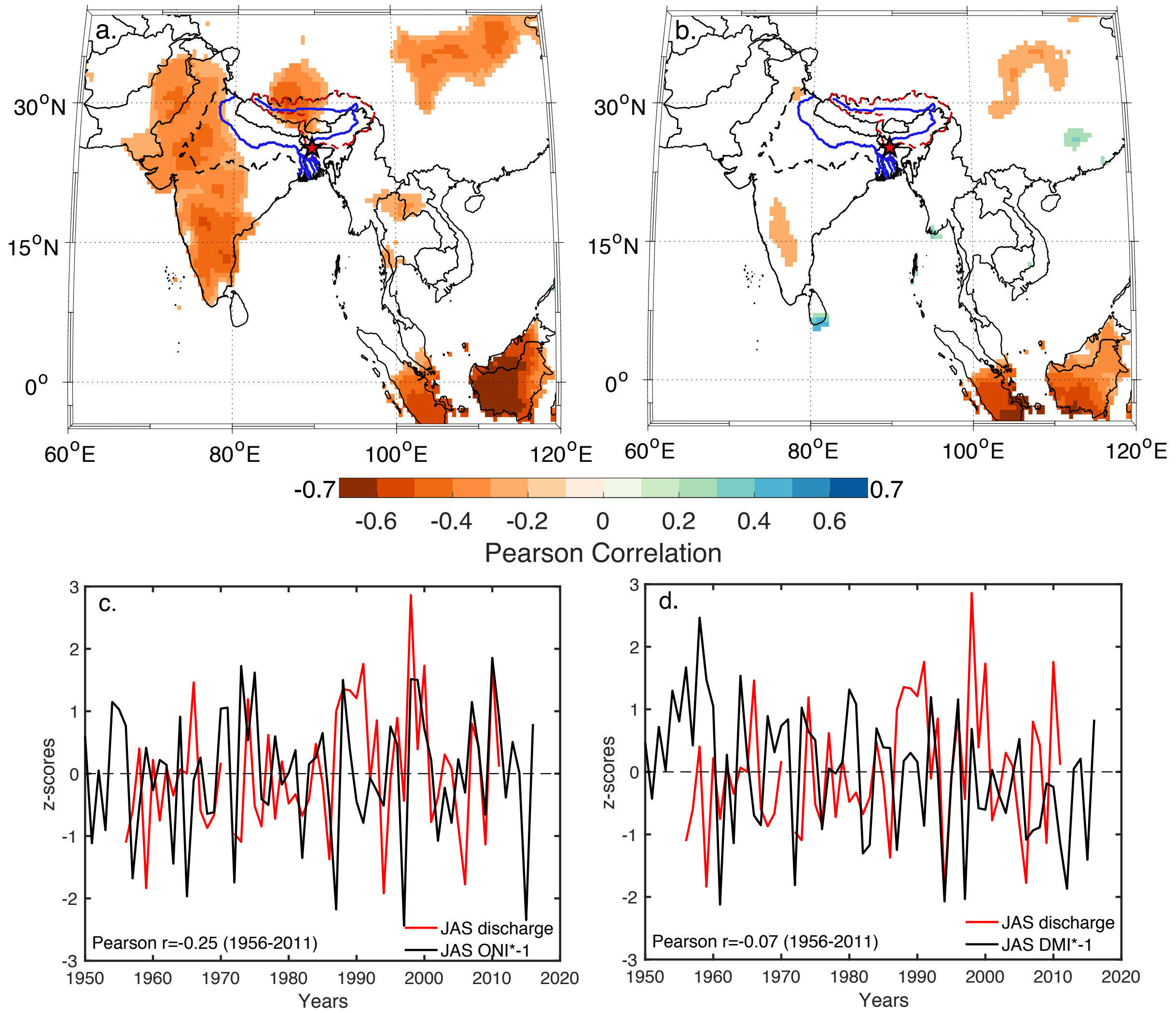

Supplementary Figure 12 Spatial correlation between mean JAS CRU Ts 4.01 precipitation and **(a)** mean JAS Oceanic Niño Index (ONI) and **(b)** mean JAS Indian Ocean Dipole (IOD) conditions based on the Dipole Mode Index (DMI) between 1950-2016 C.E. Over South Asia, correlations between JAS precipitation and ONI are the strongest over western India and Pakistan and are largely located outside the Brahmaputra basin. Correlations between DMI and regional precipitation are largely insignificant. Only correlations significant at $p<0.05$ using a 2 tailed t-test are shown in (a) and (b). The two lower panel plots show standardised anomalies of mean JAS Brahmaputra discharge at Bahadurabad plotted against standardised anomalies **(c)** JAS ONI and **(d)** JAS DMI. Neither correlation is significant at $p<0.05$ using a 2-tailed t-test, though we note that the relationship in **c.** may be non-stationarity. Note that ONI and DMI indices are multiplied by -1 in **c** and **d**. ONI data is available here: https://catalog.data.gov/dataset/climate-prediction-center-cpcoceanic-nino-index, and DMI data at this link: https://psl.noaa.gov/gcos_wgsp/Timeseries/DMI/.

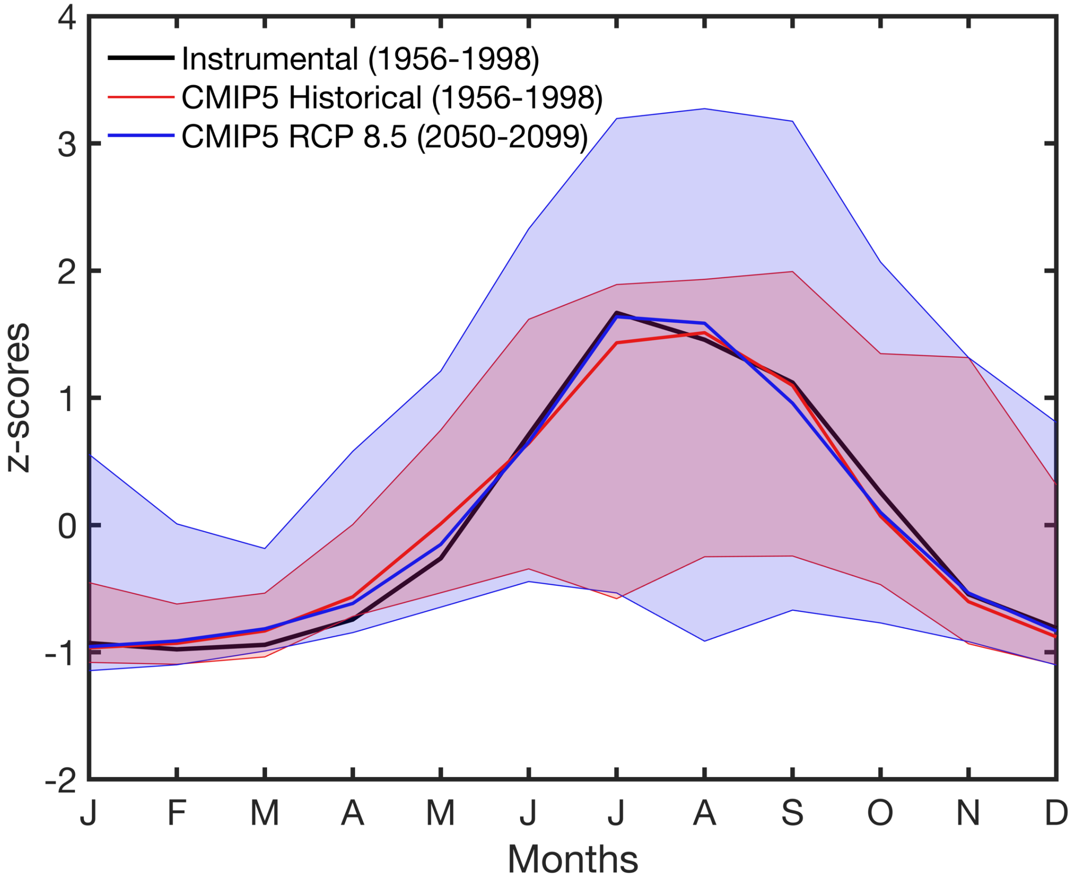

Supplementary Figure 13 Standardized anomalies of annual mean discharge of the Brahmaputra River at Bahadurabad, Bangladesh between 1956-1998 C.E. (in black) compared against standardized anomalies of annual runoff integrated over the Brahmaputra watershed upstream of Bahadurabad from the CMIP5 climate model suite between 1956-1998 C.E. from the 'historical' simulation period (multi-model ensemble median in red) and 2050-2099 C.E. from the RCP8.5 simulation (multi-model ensemble median in blue). The shaded envelope represents the 10th and 90th percentiles across all 42 model simulations.

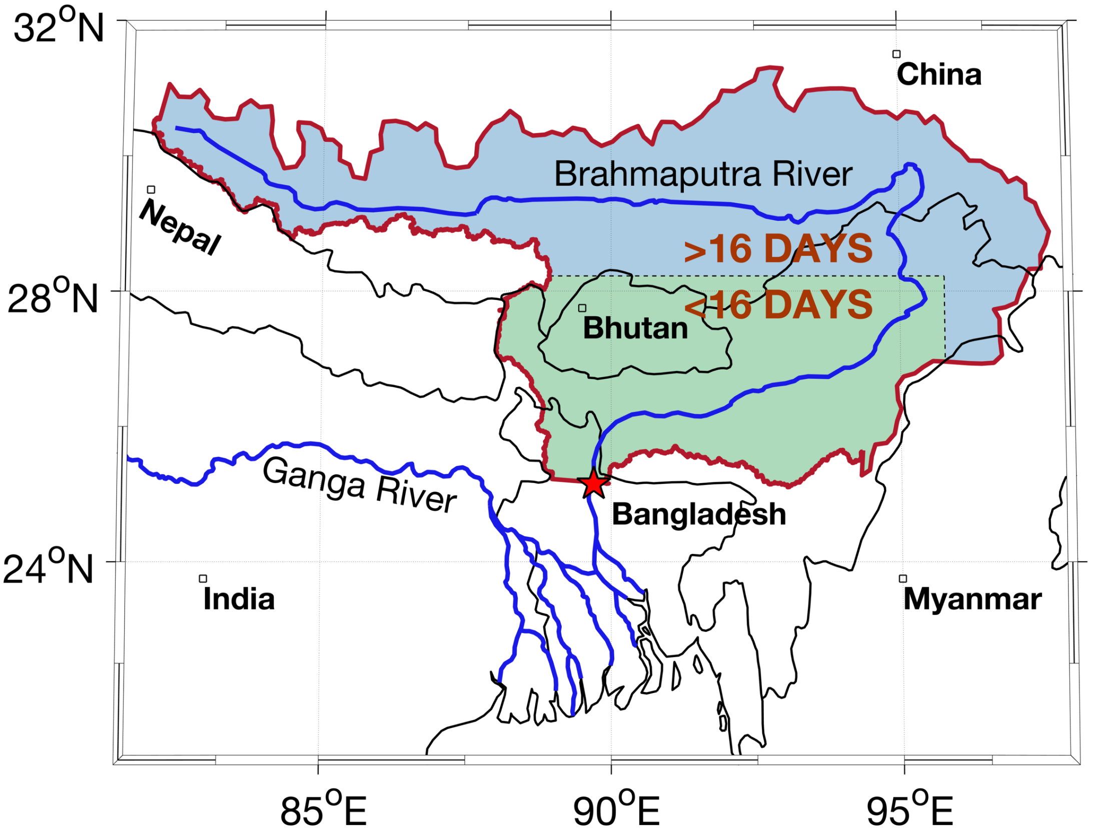

Supplementary Figure 14 Spatial partitioning of the Brahmaputra watershed into an upper and lower section (in blue and green respectively) based on the number of days discharge at a given location would take to eventually make it to the Brahmaputra River at the gauging station Bahadurabad, Bangladesh (red star). The partitioning of the watershed was based on daily isochrone maps developed by refs. - [9,10] and converted to two sections to account for that CMIP 5 runoff data was only available at a monthly resolution, and therefore and discharge at Bahadurabad arriving from the upper section of the watershed is in fact runoff from the previous month.

Supplementary Tables

Supplementary Table 1. Tree-ring predictors used in mean JAS Brahmaputra discharge reconstruction. Pearson correlation between each predictor and JAS discharge, and predictor principal component (PC1 & PC2) loadings are calculated between 1956-1998. *Lag t+1 predictors. +The two chronologies from Myanmar are new series developed by co-authors. TRW - Tree Ring Width, LWW - Late Wood Width.

	**Country**	**Site**	**Species**	**Start Year**	**End Year**	**Lat (°)**	**Lon (°)**	**Pearson ρ**	**PC1 loading**	**PC2 loading**	**Distance from watershed (km)**	**Reference**
1+	Myanmar	Chin Hills	*Pinus kesiya, TRW*	1695	2013	21.22	94.02	0.26	0.11	0.55	443	-
2+	Myanmar	Chin Hills	*Pinus kesiya, LWW*	1695	2013	21.22	94.02	0.46	0.47	0.51	443	-
3	Nepal	Chardung	*Abies spectabilus*	1689	1998	27.17	86.42	0.35	0.56	0.11	157	Cook et al. 2003[11]
4*	China	Xinlong	*Abies forrestii*	1663	2007	30.87	100.28	0.49	0.44	0.5	329	Cook et al. 2013[12]
5	Bhutan	Ghasa	*Juniperus recurva*	1660	2006	27.92	89.75	0.38	0.42	-0.3	within	Cook et al. 2010[4]; 2011[13]
6	Bhutan	Laya	*Larix griffithiana*	1644	2006	27.98	89.75	0.33	0.63	-0.22	within	Cook et al. 2010[4]; 2011[14]
7	Bhutan	Chele La	*Larix griffithiana*	1620	2005	27.38	89.32	0.53	0.69	0.24	within	Cook et al. 2010[4]; 2011[15]
8	Nepal	Lamite Bhajyung	*Abies spectabilus*	1561	1999	27.48	87.90	0.47	0.58	0.09	16	Krusic, 2005[16]
9*	China	Hebei Low	*Juniperus przewalskii*	1520	2002	34.78	100.82	0.53	0.89	-0.15	669	Cook et al. 2013[12]
10	China	Shangri La	*Abies forrestii*	1516	2007	27.62	99.80	0.31	0.41	0.43	244	Cook et al, 2010[4]; Wright et al, 2011[17]
11	Nepal	Eastern Nepal	*Abies spectabilus*	1509	1999	27.73	87.20	0.38	0.44	0.09	85	Krusic, 2005[18]
12*	China	Maxiong Valley	*Abies forrestii*	1509	2006	29.15	99.93	0.34	0.09	0.71	229	Li et al 2017[19]
13	Nepal	Yalung Khola	*Tsuga dumosa*	1500	1999	27.83	88.02	0.39	0.49	-0.02	14	Krusic, 2005[20]
14*	China	Hebei High	*Juniperus przewalskii*	1500	2002	34.78	100.82	0.29	0.55	-0.32	669	Cook et al. 2013[12]
15*	Bhutan	Dhur	*Juniperus recurva*	1462	2014	27.72	90.68	-0.30	-0.45	0.1	within	Krusic et al. 2015[21]
16	Nepal	Dobini Danda	*Juniperus recurva*	1445	1998	27.43	86.20	0.53	0.78	-0.12	161	Cook et al. 2003[11]
17	Nepal	Bhule Pokari	*Juniperus recurva*	1417	1998	27.42	86.27	0.42	0.59	-0.2	162	Cook et al. 2003[11]; Krusic & Cook 2002[22]
18*	China	Maxiong Valley All	*Abies forrestii*	1380	2007	29.15	100.00	0.29	0.14	0.82	235	Li et al 2017[19]
19*	China	Hebei Median	*Juniperus przewalskii*	1310	2002	34.78	100.82	0.41	0.7	-0.22	669	Cook et al. 2013[12]
20*	China	Zaduo	*Juniperus przewalskii*	1290	2006	32.67	95.72	0.40	0.48	-0.21	229	Cook et al. 2013[12]
21*	China	ZD31	*Juniperus spp.*	1290	2006	32.67	95.72	0.40	0.48	-0.21	229	Cook et al. 2013[12]
22*	China	QML-ZD31	*Juniperus spp.*	1290	2006	32.73	95.83	0.29	0.47	-0.41	238	Cook et al. 2013[12]
23*	China	Central Tibet	*Juniperus tibetica*	1285	2008	29.35	92.00	0.27	0.13	0.5	within	Cook et al. 2013[12]
24*	Nepal	Eastern Nepal	*Tsuga dumosa*	1260	1999	27.45	87.00	-0.30	-0.2	-0.35	103	Cook et al. 2013[12]
25*	China	TDC	*Juniperus przewalskii*	1130	2002	35.07	100.35	0.46	0.71	-0.3	665	Cook et al. 2013[12]
26	China	Maquina-A	*Juniperus przewalskii*	1082	2001	35.07	100.35	-0.32	-0.1	-0.47	665	Gou et al. 2007[23] Li et al 2017[19]
27	China	Maquina-C	*Juniperus przewalskii*	1082	2001	35.07	100.35	-0.38	-0.33	-0.38	665	Gou et al. 2007[23] Li et al 2017[19]
28*	China	Qamdo	*Juniperus tibetica*	449	2004	31.12	97.03	0.34	0.31	0.27	140	Cook et al. 2013[12]

Supplementary Table 2. List of CMIP5 models[24] used in Brahmaputra discharge simulations and in the estimates of global mean annual temperature change. We used model and its respective scenario run only if it extended through both the historical (1850-2005) and RCP8.5 (2006-2099) simulation period. For each model we first calculated the median discharge projection across ensemble members within each model, and only then calculated the median and interquartile range across models. This was done to ensure that each of the 20 models are represented equally in the final multi-model ensemble estimate.

	**Model Name**	**Scenarios**	**Modelling Centre**
1.	ACCESS1-0	r1i1p1	Centre for Australian Weather and Climate Research (CAWCR)
2.	ACCESS1-3	r1i1p1	
3.	bcc-csm1-1	r1i1p1	Beijing Climate Center, China Meteorological Administration (CMA)
4.	CanESM2	r1i1p1; r2i1p1; r3i1p1; r4i1p1; r5i1p1	Canadian Centre for Climate Modelling and Analysis
5.	CCSM4	r1i1p1; r2i1p1; r3i1p1; r4i1p1; r5i1p1; r6i1p1	NCAR/UCAR Community Climate System Model
6.	CNRM-CM5	r1i1p1; r2i1p1; r4i1p1; r6i1p1	Centre National de Recherches Meteorologiques / Centre Europeen de Recherche et Formation Avancees en Calcul Scientifique (CNRM/CERFACS)
7.	FIO-ESM	r2i1p1; r3i1p1; r2i1p1; r3i1p1	First Institute of Oceanography, State Oceanic Administration, China
8.	GFDL-ESM2G	r1i1p1	NOAA Geophysical Fluid Dynamics Laboratory
9.	GFDL-ESM2M	r1i1p1	
10.	GISS-E2-R	r1i1p1	NASA Goddard Institute for Space Studies
11.	inmcm4	r1i1p1	Institute for Numerical Mathematics
12.	IPSL-CM5A-LR	r1i1p1; r2i1p1; r3i1p1; r4i1p1	Institut Pierre-Simon Laplace
13.	IPSL-CM5A-MR	r1i1p1	
14.	IPSL-CM5B-LR	r1i1p1	
15.	MIROC-ESM	r1i1p1	Atmosphere and Ocean Research Institute (The University of Tokyo), National Institute for Environmental Studies, and Japan Agency for Marine-Earth Science and Technology
16.	MIROC-ESM-CHEM	r1i1p1	
17.	MIROC5	r1i1p1. r2i1p1; r3i1p1	
18.	MPI-ESM-LR	r1i1p1; r2i1p1; r3i1p1	Max Planck Institute for Meteorology (MPI-M)
19.	MRI-CGCM3	r1i1p1	Meteorological Research Institute
20.	NorESM1-M	r1i1p1	Norwegian Climate Centre (NorClim)
		**Total: 42**	

Supplementary References

1 Huffman, G. J. *et al.* The TRMM Multisatellite Precipitation Analysis (TMPA): Quasi-Global, Multiyear, Combined-Sensor Precipitation Estimates at Fine Scales. *J. Hydrometeorol.* **8**, 38-55, doi:10.1175/jhm560.1 (2007).

2 Schneider, U. *et al.* Evaluating the Hydrological Cycle over Land Using the Newly-Corrected Precipitation Climatology from the Global Precipitation Climatology Centre (GPCC). *Atmosphere* **8**, 52 (2017).

3 van der Schrier, G., Barichivich, J., Briffa, K. R. & Jones, P. D. A scPDSI-based global data set of dry and wet spells for 1901–2009. *Journal of Geophysical Research: Atmospheres* **118**, 4025-4048, doi:doi:10.1002/jgrd.50355 (2013).

4 Cook, E. R. *et al.* Asian Monsoon Failure and Megadrought During the Last Millennium. *Science* **328**, 486-489, doi:10.1126/science.1185188 (2010).

5 Chen, Y. *et al.* Precipitation variations recorded in tree rings from the upper Salween and Brahmaputra River valleys, China. *Ecol. Indicators* **113**, 106189, doi:https://doi.org/10.1016/j.ecolind.2020.106189 (2020).

6 He, M., Bräuning, A., Grießinger, J., Hochreuther, P. & Wernicke, J. May–June drought reconstruction over the past 821 years on the south-central Tibetan Plateau derived from tree-ring width series. *Dendrochronologia* **47**, 48-57, doi:https://doi.org/10.1016/j.dendro.2017.12.006 (2018).

7 Shi, C. *et al.* The response of relative humidity to centennial-scale warming over the southeastern Tibetan Plateau inferred from tree-ring width chronologies. *Clim. Dyn.* **51**, 3735-3746, doi:10.1007/s00382-018-4107-5 (2018).

8 Wang, J., Yang, B. & Ljungqvist, F. C. Moisture and Temperature Covariability over the Southeastern Tibetan Plateau during the Past Nine Centuries. *J. Clim.* **0**, null, doi:10.1175/jcli-d-19-0363.1 (2020).

9 Jian, J., Webster, P. J. & Hoyos, C. D. Large-scale controls on Ganges and Brahmaputra river discharge on intraseasonal and seasonal time-scales. *Q. J. Roy. Meteorol. Soc.* **135**, 353-370, doi:10.1002/qj.384 (2009).

10 Webster, P. J. *et al.* Extended-Range Probabilistic Forecasts of Ganges and Brahmaputra Floods in Bangladesh. *Bull. Am. Meteorol. Soc.* **91**, 1493-1514, doi:10.1175/2010bams2911.1 (2010).

11 Cook, E. R., Krusic, P. J. & Jones, P. D. Dendroclimatic signals in long tree-ring chronologies from the Himalayas of Nepal. *Int. J. Climatol.* **23**, 707-732, doi:10.1002/joc.911 (2003).

12 Cook, E. R. *et al.* Tree-ring reconstructed summer temperature anomalies for temperate East Asia since 800 CE. *Clim. Dyn.* **41**, 2957-2972 (2013).

13 Cook, E. R., Krusic, P. J. & Dukpa, D. NOAA/WDS Paleoclimatology - Cook - Gasa-1 - JURE - ITRDB BT019. *NOAA National Centers for Environmental Information*, doi:https://doi.org/10.25921/dya4-4405. (2011).

14 Cook, E. R., Krusic, P. J. & Dukpa, D. NOAA/WDS Paleoclimatology - Cook - Laya-1 - LAGR - ITRDB BT016. *NOAA National Centers for Environmental Information*, doi:https://doi.org/10.25921/6dqw-ra92 (2011).

15 Cook, E. R., Krusic, P. J. & Dukpa, D. NOAA/WDS Paleoclimatology - Cook - Chele La - LAGR - ITRDB BT017. *NOAA National Centers for Environmental Information*, doi:https://doi.org/10.25921/6qya-d315 (2011).

16 Krusic, P. J. NOAA/WDS Paleoclimatology - Krusic - Lamite Bhajyung - ABSB - ITRDB NEPA027. *NOAA National Centers for Environmental Information*, doi:https://doi.org/10.25921/evfb-nd64 (2005).

17 Wright, W., Li, J., Fang, K., Tao, Y. & Cook, E. R. NOAA/WDS Paleoclimatology - Wright - Shangri La - ABFO - ITRDB CHIN026. *NOAA National Centers for Environmental Information*, doi:https://doi.org/10.25921/rys9-y012 (2011).

18 Krusic, P. J. NOAA/WDS Paleoclimatology - Krusic - Rachel's Death - ABSB - ITRDB NEPA036. *NOAA National Centers for Environmental Information*, doi:https://doi.org/10.25921/x2zf-gs03 (2005).

19 Li, J. *et al.* Moisture increase in response to high-altitude warming evidenced by tree-rings on the southeastern Tibetan Plateau. *Clim. Dyn.* **48**, 649-660, doi:10.1007/s00382-016-3101-z (2017).

20 Krusic, P. J. NOAA/WDS Paleoclimatology - Krusic - Yalung Khola - TSDU - ITRDB NEPA042. *NOAA National Centers for Environmental Information*, doi:https://doi.org/10.25921/r737-3v36 (2005).

21 Krusic, P. J. *et al.* Six hundred thirty-eight years of summer temperature variability over the Bhutanese Himalaya. *Geophys. Res. Lett.* **42**, 2988-2994, doi:10.1002/2015gl063566 (2015).

22 Krusic, P. J. & Cook, E. R. NOAA/WDS Paleoclimatology - Krusic - Bhule Pokari - JURE - ITRDB NEPA010. *NOAA National Centers for Environmental Information*, doi:https://doi.org/10.25921/xcp5-7a23 (2002).

23 Gou, X. *et al.* Streamflow variations of the Yellow River over the past 593 years in western China reconstructed from tree rings. *Water Resour. Res.* **43**, doi:10.1029/2006wr005705 (2007).
24 Taylor, K. E., Stouffer, R. J. & Meehl, G. A. An Overview of CMIP5 and the Experiment Design. *Bull. Am. Meteorol. Soc.* **93**, 485-498, doi:10.1175/bams-d-11-00094.1 (2012).
